# Area-dependent change of response in the rat’s inferior colliculus to intracochlear electrical stimulation following neonatal cochlear damage

**DOI:** 10.1038/s41598-019-41955-y

**Published:** 2019-04-04

**Authors:** Miyako Hatano, Jack B. Kelly, Huiming Zhang

**Affiliations:** 1 Department of Otolaryngology-Head and Neck Surgery, Kanazawa University, Kanazawa, 920-8640 Ishikawa Japan; 2 Department of Neuroscience, Carleton University, Ottawa, Ontario K1S 5B6 Canada; 3 Department of Biological Sciences, University of Windsor, Windsor, Ontario N9B 3P4 Canada

## Abstract

To understand brain changes caused by auditory sensory deprivation, we recorded local-field potentials in the inferior colliculus of young adult rats with neonatal cochlear damage produced by systemic injections of amikacin. The responses were elicited by electrical stimulation of the entire cochlea and recorded at various locations along a dorsolateral-ventromedial axis of the inferior colliculus. We found that hair cells were completely destroyed and spiral ganglion neurons were severely damaged in the basal cochleae of amikacin-treated animals. Hair cells as well as spiral ganglion neurons were damaged also in the middle and apical areas of the cochlea, with the damage being greater in the middle than the apical area. Amplitudes of local-field potentials were reduced in the ventromedial inferior colliculus, but enhanced in the dorsolateral inferior colliculus. Latencies of responses were increased over the entire structure. The enhancement of responses in the dorsolateral inferior colliculus was in contrast with the damage of hair cells and spiral ganglion cells in the apical part of the cochlea. This contrast along with the overall increase of latencies suggests that early cochlear damage can alter neural mechanisms within the inferior colliculus and/or the inputs to this midbrain structure.

## Introduction

Sensory deprivation caused by a cochlear damage can lead to changes in the central auditory system including reorganization of the system^1–6^. These central changes can substantially affect the processing of acoustic information. The effect of cochlear damage is particularly pronounced if it occurs prior to or within the critical period of hearing development. In young human subjects, such sensory deprivation can affect language acquisition^7,8^. Deaf children who receive cochlear implants at an earlier age tend to have better speech perception and language skills than those who receive implants at a later age^9–12^. A delay in cochlear implantation can reduce the effectiveness of the treatment. This age-dependent effect of cochlear implantation is at least partly due to plastic changes in the central auditory system^13,14^.

Cochlear damage can be caused by aminoglycoside antibiotics including amikacin. These drugs can result in extensive destruction of inner and outer hair cells (IHCs and OHCs), spiral ganglion neurons (SGNs), and other cells in the cochlea and lead to profound hearing loss^15^. Amikacin typically affects high frequency hearing first^16–19^. Low frequency hearing is also affected as the drug effect progresses. The drug applied at a high dose and over a long duration can completely or almost completely abolish the sensitivity to sounds in rats and other mammals including humans^17,18,20^.

The present study used the rat as an animal model to examine the effect of neonatal cochlear damage caused by amikacin on neural activity in the inferior colliculus (IC), a key midbrain auditory processing center^21^. The IC receives inputs from all the major brainstem and forebrain auditory structures and provides outputs to the auditory thalamus as well as major brainstem auditory structures^22–26^. The IC can be subdivided into the central nucleus, lateral cortex, ventrolateral nucleus, and the dorsal cortex^27^. Neurons in the central nucleus are tonotopically organized, with those sensitive to low frequencies located in the dorsolateral region and those sensitive to high frequencies located in the ventromedial region^28^. As the IC plays a key role in neural processing and has extensive connections with other auditory neural processing centers, a change of response in the IC can severely affect hearing.

It is well established that neural activity in the IC can be affected by auditory sensory deprivation. Partial cochlear damage caused by amikacin or an intense noise can lead to functional reorganizations including alteration of the tonotopic map in the IC^6,29,30^. Cochlear damage caused by an intense noise or a drug such as carboplatin can lead to an enhancement of population neural activity in the IC^4,31–34^. In these previous studies, responses in the IC were evoked using acoustic stimulation and were dependent on sensory transduction in receptor cells. Evaluation of central contributions to the responses in the IC could be complicated by damage to auditory receptor cells caused by a drug or an intense noise. Direct activation of SGNs by intracochlear electrical stimulation can help the evaluation of the central contribution^35^.

In the present study, we electrically stimulated the entire cochlea to elicit neural responses in the IC of young adult rats that had received daily systemic treatments of amikacin over postnatal days 7 through 16. We found that a local-field potential (LFP) in response to intracochlear stimulation was enhanced in the dorsolateral IC and reduced in the ventromedial IC in drug-treated animals. In contrast, IHCs and OHCs as well as SGNs were damaged in all regions of the cochlea. This contrast suggested that peripheral damage caused by the drug resulted in functional reorganization in the central auditory pathway involving the IC.

## Results

LFPs were recorded from the IC in 7 normal and 7 amikacin-treated rats. Of these animals, survival rates of IHC and OHC and densities of SGN were evaluated in 4 normal rats and 4 amikacin-treated rats. Cochlear anatomy was qualitatively examined in the remaining 3 normal and 3 amikacin-treated rats.

### Effects of amikacin on peripheral auditory structures

**Figure 1:**
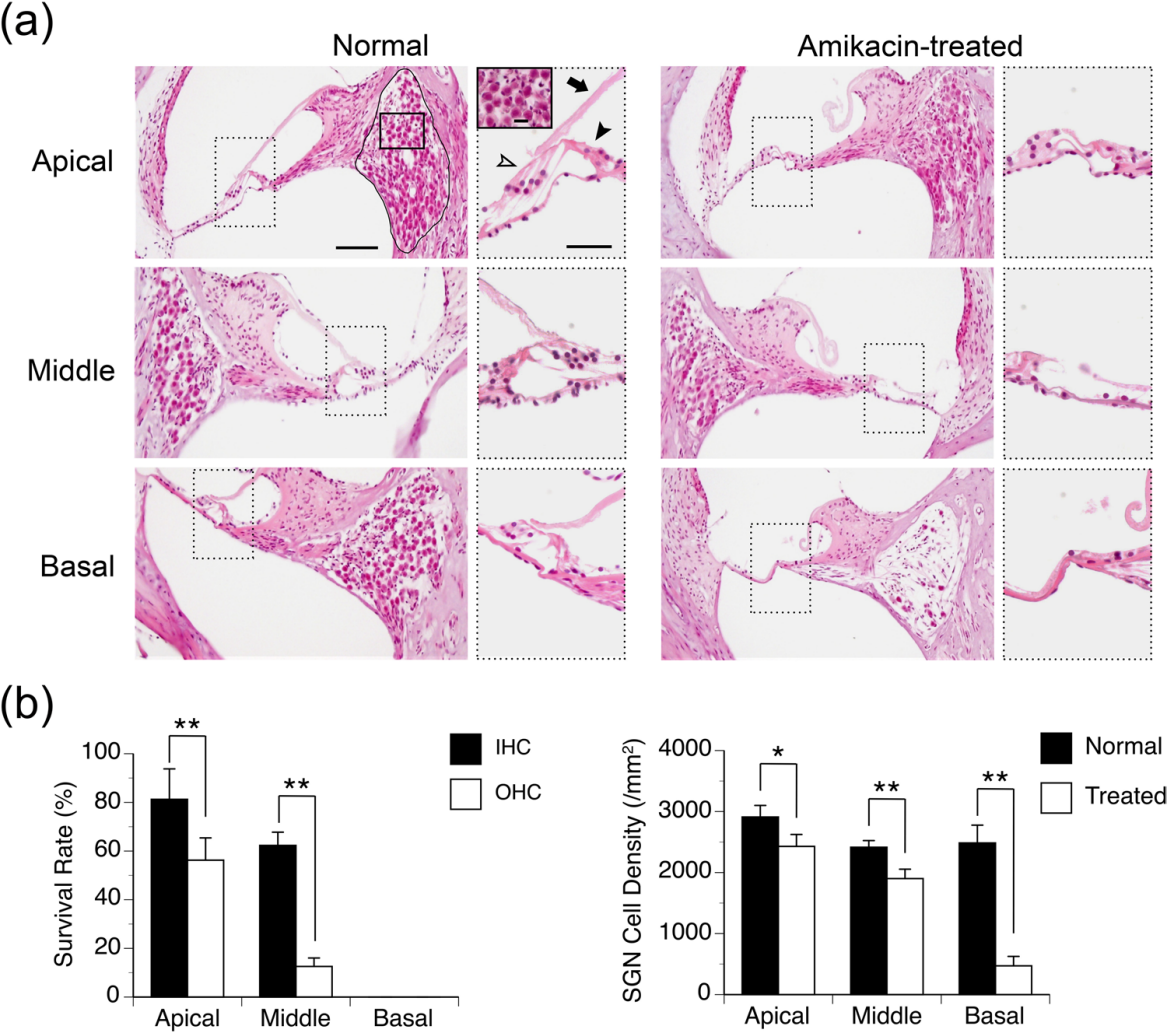
Effects of amikacin on the peripheral auditory system. (**a**) Photomicrographs of cross sections of the cochlea from a normal (left panels) and an amikacin-treated (right panels) animal. In each of the low-magnification images, a dotted-line rectangle shows where a high-magnification image of the Organ of Corti (the inset with a dotted outline) was taken. In the low-magnification image of the top-left panel, a solid enclosed curve shows the outline of the Rosenthal’s canal. A solid-line rectangle shows the location where a high-magnification image of SGNs (the inset with a solid outline) was taken. In the inset of the top-left panel, “➤” points towards an IHC while “” points towards OHCs. “➡” points toward the tectorial membrane. Scale bars: 100 μm in the low-magnification image, 50 μm in the inset showing the Organ of Corti, and 20 μm in the inset showing SGNs. **(b)** Group results showing effects of amikacin treatments on the number of hair cells (left panel, results from 4 amikacin-treated animals) and SGNs (right panel, results from 4 normal and 4 amikacin-treated animals). Error bar indicates standard error of the mean. “*” and “**” Indicate statistical significance at levels of p < 0.05 and p < 0.001, respectively.

Treatment with amikacin severely damaged the peripheral auditory system in all of the 7 rats in the experimental group. For the example shown in the right column of Fig. 1a, the IHC in the basal turn was lost while those in the middle and apical turns displayed certain degrees of degeneration. All of the OHCs in the basal turn were lost. The OHCs in the middle turn were severely damaged while those in the apical turn displayed some degeneration. Thus, the damage of hair cells was more severe in the basal than the apical part of the cochlea. Furthermore, OHCs were affected more than IHCs by the drug. These qualitative observations were supported by survival rates of hair cells (Fig. 1b left panel). A logistic regression analysis revealed that the survival rate of OHC was significantly lower than that of the IHC at the apical (odds ratio = 0.30; *p* < 0.001) and middle turns (odds ratio = 0.09; *p* < 0.001). In addition to the loss of hair cells, other changes such as collapse of the tectorial membrane were observed in the cochleae of amikacin-treated animals.

Amikacin caused extensive loss of SGNs in the apical, middle, and basal parts of the cochlea, as shown by the example in Fig. 1a right column. The loss was greatest in the basal turn. The density of SGNs was quantified in four normal rats and four amikacin-treated rats (Fig. 1b right panel). A two-way ANOVA analysis based on these quantified results supported the observation that drug treatments caused a significant reduction in cell density (F (1, 18) = 182.54, p < 0.001). The reduction was different across apical, middle, and basal parts of the cochlea (F (2, 18) = 77.50, p < 0.001). Furthermore, there was a difference in the location by treatment interaction (F (2, 18) = 38.03, *p* < 0.001). An independent samples t-test indicated that the density of SGN was significantly reduced at all the three regions of the cochlea (apical: t (6) = 3.52, p = 0.013; middle: t (6) = 7.13, p < 0.001; basal: t (6) = 12.17, p < 0.001).

### Intracochlear stimulation-evoked LFPs in the IC of normal animals

**Figure 2:**
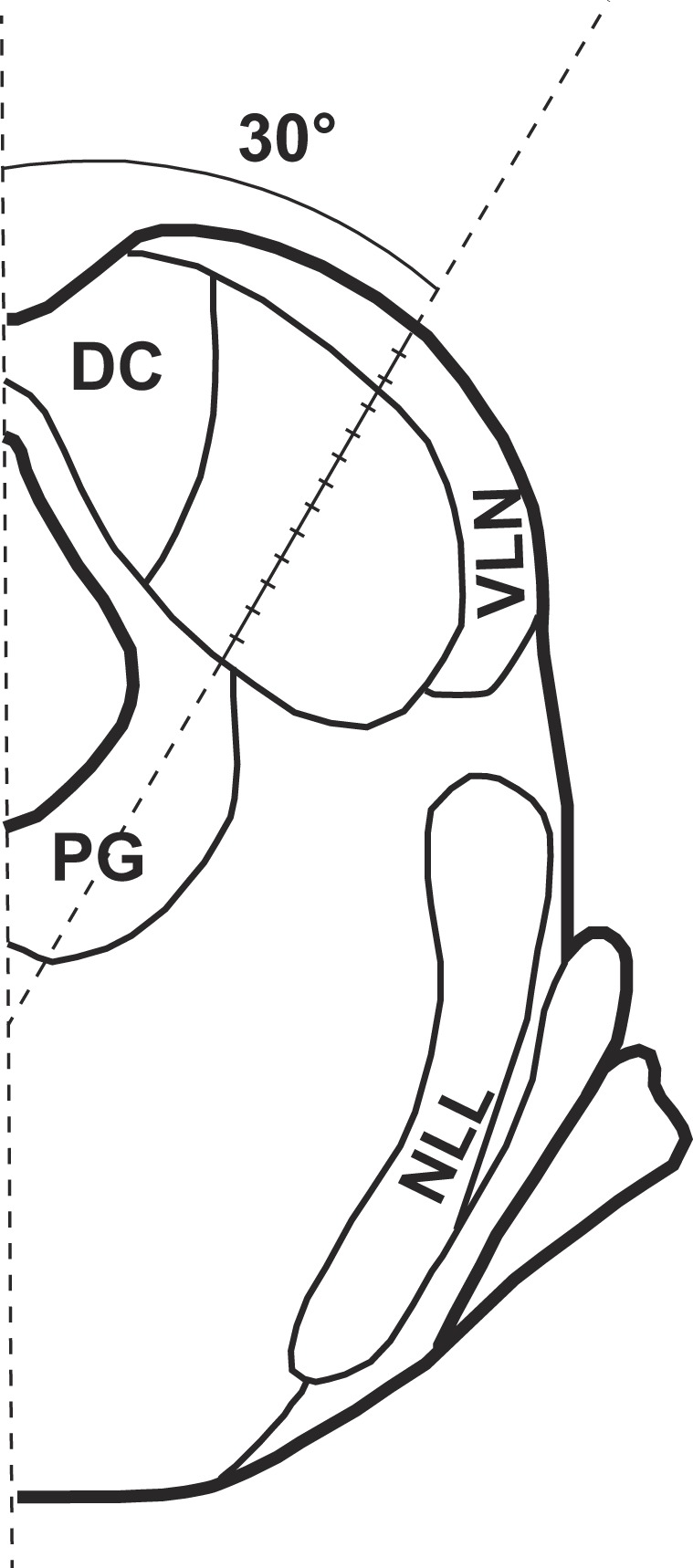
Orientation of a recording electrode and the locations where LFPs in response to intracochlear electrical stimulation were recorded in the IC. The electrode had a 30° angle in reference to the midsagittal plane. The solid part of the tilted line indicates the extent within the IC. Hash marks indicate locations of recording. The distance between two adjacent hash marks is 200 μm. The maximum distance from the dorsolateral to the ventromedial edge of the IC was measured along an axis with the same orientation as that of the recording electrode. PG: periaqueductal gray; NLL: nucleus of the lateral lemniscus; DC: dorsal cortex of the inferior colliculus; VLN: ventrolateral nucleus of the inferior colliculus.

In each rat, an LFP in response to electrical stimulation of the left cochlea was recorded from the right IC at 12 loci at depths of 200 to 2400 µm from the surface of the structure in 200 µm steps (Fig. 2). These loci covered both high and low frequency regions of the central nucleus of the IC.

**Figure 3:**
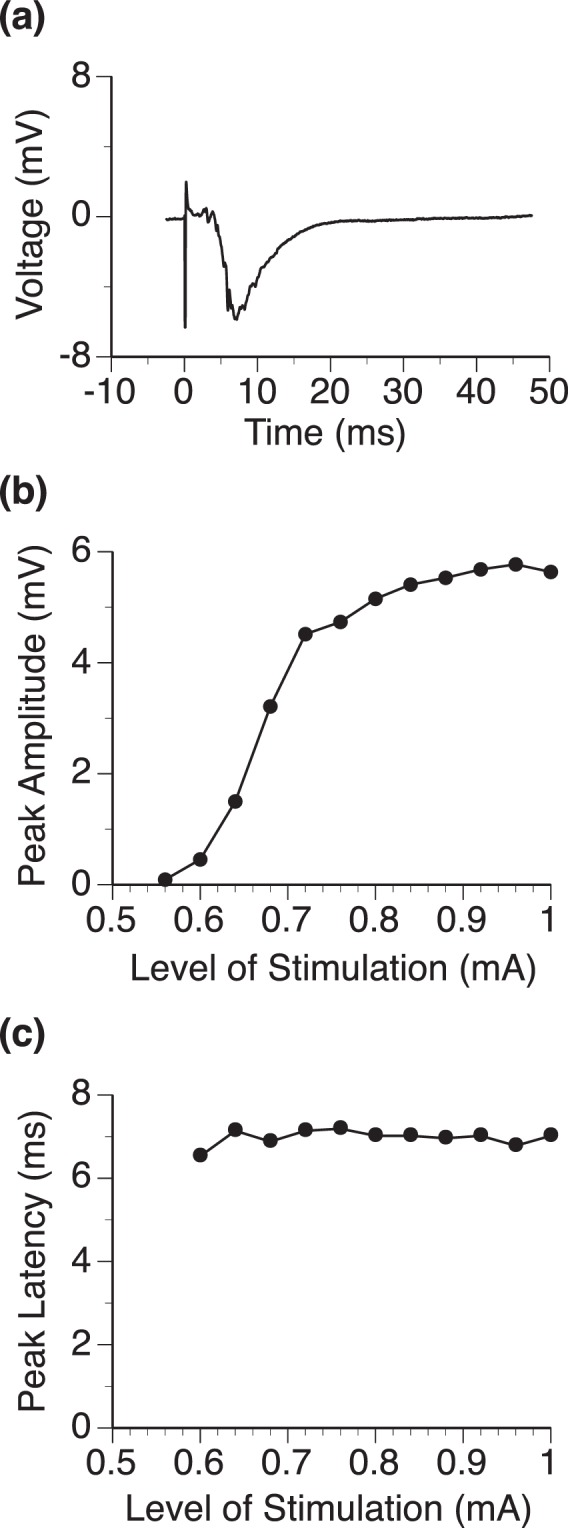
Typical LFP recorded at one single location (depth = 1.2 mm) in the IC of a normal rat. (**a**) Waveform of an LFP response. **(b)** Line chart showing the relationship between the amplitude of the negative peak of an LFP and the level of stimulation of the cochlea. **(c)** Line chart showing the relationship between the latency of the negative peak of an LFP and the level of stimulation of the cochlea.

An LFP evoked by intracochlear electrical stimulation typically had a dominant negative peak (Fig. 3a). Although the overall shape of the waveform was not dependent on the level of stimulation, the amplitude of the negative peak was monotonically increased and the peak latency was minimally affected when the level of stimulation was increased (Fig. 3b,c).

**Figure 4:**
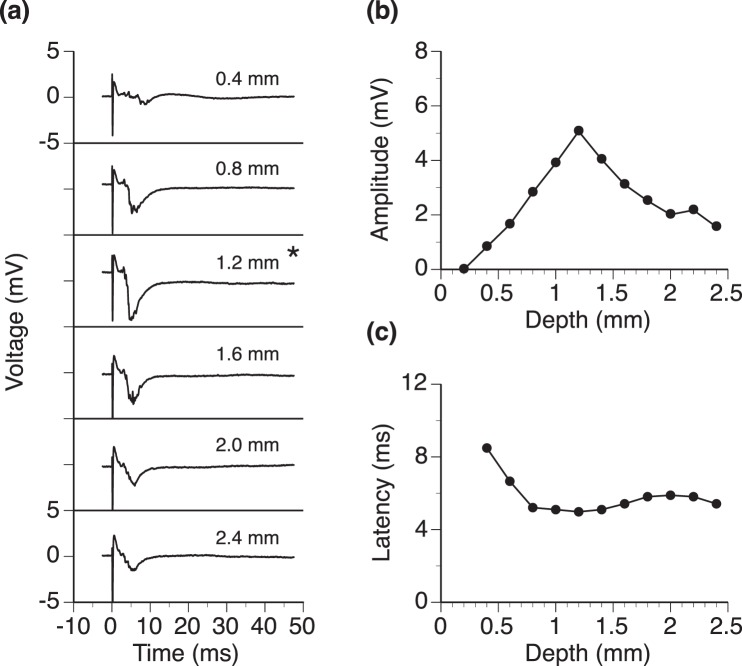
LFPs recorded at different locations in a normal rat’s IC along an axis at 30° in reference to the midsagittal plane. (**a**) Waveforms of LFP responses. **(b)** Line chart showing the relationship between the amplitude of the negative peak of an LFP and depth of recording. **(c)** Line chart showing the relationship between the latency of the negative peak of an LFP and depth of recording. Responses at all locations were obtained when the intracochlear stimulation was delivered at 0.77 mA. Although recordings were obtained at 12 loci in the IC, only 6 waveforms are shown in **(a)** for visual clarity. In **(a)**, the number in the upper right corner of each panel indicates the depth at which a waveform was recorded. * Indicates the depth at which the strongest response was observed. The same scale is used for making all of the six panels of **(a)**. Labels of the y-axis are only indicated for the top and bottom panels for visual clarity.

In response to the same intracochlear stimulation, LFPs obtained at different loci of the IC had the same overall shape but different amplitudes and latencies (Fig. 4a). The amplitude was smallest at the dorsolateral and ventromedial edges (i.e., depth of 0.2 mm or 2.4 mm) and largest at the center (i.e., depth of 1.2 mm) of the structure (Fig. 4b). The latency of the negative peak was reduced over the first 0.4 mm along a dorsolateral-ventromedial axis and was relatively constant over the rest of the axis (Fig. 4c).

**Figure 5:**
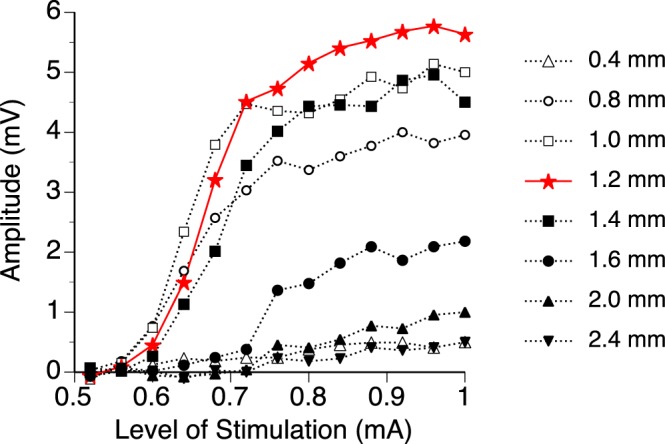
Relationships between the amplitude of the negative peak of an LFP and the level of cochlea stimulation. Relationships were studied at 12 different locations along an axis 30° in reference to the midsagittal plane. Only 8 curves are shown for visual clarity. The red solid-line curve (i.e., the one with the largest saturated level) was obtained at a depth of 1.2 mm, while the 7 dotted-line curves were obtained at other depths. Results show in Figs 3 and 5 are from the same normal rat.

For the negative peak, the amplitude by level of stimulation functions obtained at different loci of recording shared the same overall shape (Fig. 5). Amplitudes reached a saturated value when intracochlear stimulation reached a high level (0.92 mA for this example). The saturated value was the largest when the depth was 1.2 mm.

### Effect of amikacin on intracochlear stimulation-evoked LFPs in the IC

**Figure 6:**
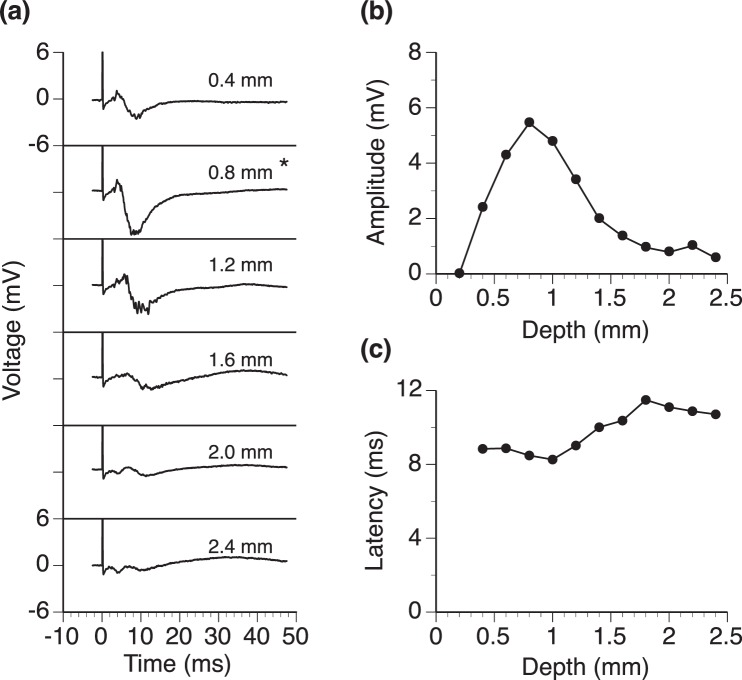
LFPs recorded at different locations in the IC of an amikacin-treated rat. (**a**) Waveforms of LFP responses along an axis at 30° in reference to the midsagittal line. (**b**) Line chart showing the relationship between the amplitude of the negative peak of an LFP and depth of recording. (**c**) Line chart showing the relationship between the latency of the negative peak of an LFP and depth of recording. Responses at all locations were obtained when the level of the intracochlear stimulation was at 0.51 mA. In **(a)**, the number in the upper right corner of each panel indicates the depth at which a waveform was recorded. * Indicates the depth at which the strongest response was observed. The same scale is used for making all of the six panels shown in **(a)**. Labels of the y-axis are only indicated for the top and bottom panels for visual clarity.

An LFP recorded from an amikacin-treated animal had the same basic shape as that recorded from a normal animal (Fig. 6a). The negative peak of an LFP displayed a depth-dependence in amplitude (Fig. 6b). For this example, the amplitude increased along the dorsolateral-ventromedial axis until the largest amplitude was reached at a depth of 0.8 mm then decreased precipitously over the rest of the axis. The latency of the negative peak increased over the depths from 1.0 mm to 1.8 mm (Fig. 6c).

**Figure 7:**
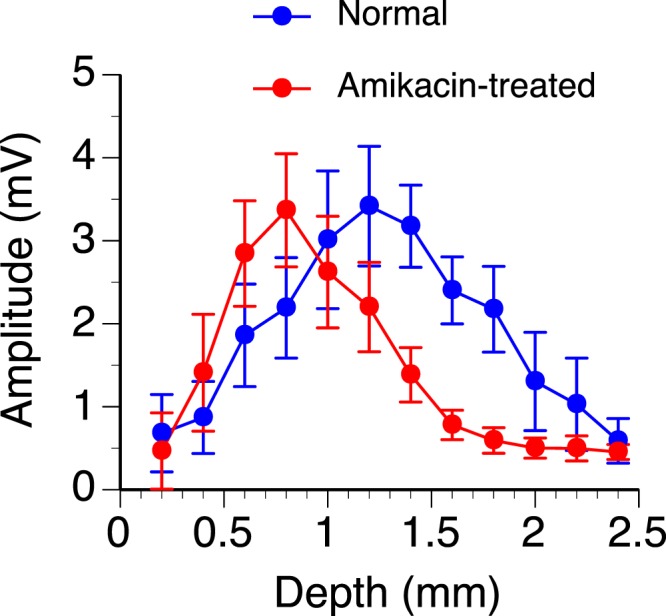
Group results showing the dependence of the absolute amplitude of the negative peak of an LFP on the depth of recording in normal (n = 7) and amikacin-treated (n = 7) rats. Blue and red curves indicate results from normal and amikacin-treated animals, respectively. Error bar indicates standard error of the mean. The curves for normal and amikacin-treated animals have similar bell shapes and maximum amplitudes (3.41 ± 0.72 mV in normal vs. 3.37 ± 0.68 mV in treated animals) but different peak locations (0.8 mm in normal vs. 1.2 mm in treated animals).

The dependence of the amplitude of the negative peak on the depth of recording was studied in all of the control and amikacin-treated animals. Group results indicated that the largest LFP for normal rats was at the center of the IC (viz. a depth of 1.2 mm) while that for amikacin-treated rats was at a dorsolateral location of the IC (viz. a depth of 0.8 mm) (Fig. 7). A two-way ANOVA analysis revealed a significant effect of depth regardless of treatment condition (F (11, 144) = 7.50, P < 0.001). It also revealed an effect of treatment (F (1,144) = 4.57, p = 0.034), which reflected a shift of peak location rather than a change in the maximum amplitude of response (3.41 ± 0.72 mV in normal vs. 3.37 ± 0.68 mV in amikacin-treated animals). Furthermore, the analysis revealed an effect of depth by treatment interaction (F (11, 144) = 2.51, p = 0.006).

**Figure 8:**
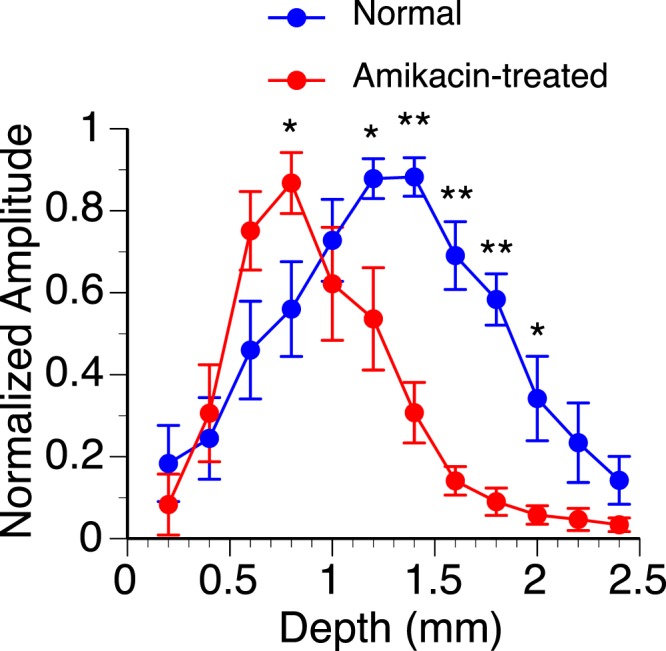
Group results showing the dependence of the normalized amplitude of the negative peak of an LFP on the depth of recording in normal (n = 7) and amikacin-treated (n = 7) rats. Blue and red lines indicate results from normal and amikacin-treated animals, respectively. Error bar indicates standard error of the mean. The curves for normal and amikacin-treated animals have similar bell shapes and maximum amplitudes but different peak locations (0.8 mm in normal vs. 1.2 mm in treated animals). An independent samples t-test revealed that the peak amplitude between normal and amikacin-treated rats was significantly different at the depths of 0.8 mm (t(12) = 2.24, p = 0.045), 1.2 mm (t(12) = 2.55, p = 0.013), 1.4 mm (t(12) = 6.57, p < 0.001), 1.6 mm (t(12) = 6.12, p < 0.001), 1.8 mm (t(12) = 6.94, p < 0.001), 2.0 mm (t(12) = 2.70, p = 0.010). * Indicates statistical significance at the level of p < 0.05. ** Indicates statistical significance at the level of p < 0.001.

Variation existed in the amplitude of response from one individual rat to another regardless of treatment condition. Despite such variation, the overall shape of an amplitude-depth curve and the best depth of response were similar across different animals within each group of rats. Thus, we normalized amplitudes of responses obtained at all depths of recording against the maximum amplitude of response within individual animals to facilitate the evaluation of the effect of drug treatment (Fig. 8). An ANOVA test on normalized amplitudes of responses revealed a significant effect of depth (F (11, 144) = 16.24, p < 0.001), drug treatment (F (1, 144) = 25.16, p < 0.001), and interaction between depth and treatment conditions (F (11, 144) = 6.07, p < 0.001). Furthermore, an independent samples t-test indicated the drug significantly changed the peak amplitude at depths between 0.8 and 2.0 mm except for 1.0 mm (see Fig. 8 caption for statistical results). The mean amplitude of responses for treated animals was greater than normal at 0.8 mm but less than normal at depths between 1.2 and 2.0 mm. These results indicated that the best depth of LFP response was shifted towards a dorsolateral region of the IC in amikacin-treated animals.

**Figure 9:**
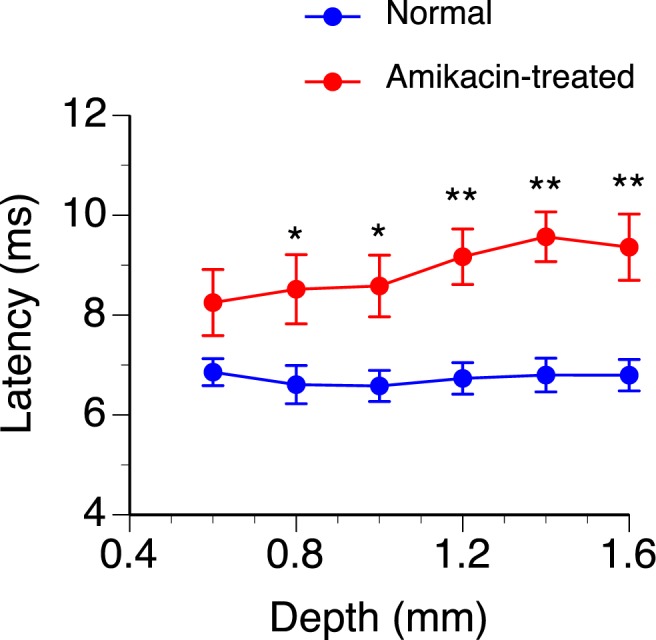
Group results showing dependences of the latency of the negative peak of an LFP on the depth of recording in normal (n = 7) and amikacin-treated (n = 7) rats. Blue and red lines indicate results from normal and amikacin-treated animals, respectively. Error bar indicates standard error of the mean. An independent samples t-test revealed that the latency of the negative peak between normal and amikacin-treated rats was significantly different at the depths of 0.8 mm (t(12) = 2.30, p = 0.042), 1.0 mm (t(12) = 2.90, p = 0.013), 1.2 mm (t(12) = 3.81, p = 0.002), 1.4 mm (t(12) = 4.60, p = 0.001), 1.6 mm (t(12) = 3.67, p = 0.004). * Indicates statistical significance at the level of p < 0.05. ** Indicates statistical significance at the level of p < 0.005.

The latency of the negative peak of LFP was examined at different loci of recording for normal and amikacin-treated animals. The latency at each locus was determined using the maximum response obtained at this locus. A measurement of latency could be reliably obtained from each of the normal and amikacin-treated animals at depths between 0.6 and 1.6 mm. Within this range, the mean latency was longer in amikacin-treated than normal animals (Fig. 9). A two-way ANOVA analysis revealed a significant effect of drug treatment (F (1, 69) = 57.49, p < 0.001), but not depth (F (5, 69) = 0.66, p = 0.653) or depth by treatment interaction (F (5, 69) = 0.512, p = 0.766). Independent samples t-tests indicated that the effect of the drug was significant at depths between 0.8 and 1.6 mm (see Fig. 9 caption for statistical results). Thus, statistical analyses indicated that the drug caused an overall but not depth-dependent increase in the latency of response.

### Effects of amikacin on the size of the IC

To determine whether amikacin affected the best location of the LFP response by altering the physical size of the IC, the size of the structure was measured along an axis that was 30° relative to the midsagittal plane (Fig. 2) for each of the animals in the normal and amikacin-treated groups. No difference was found between normal (2358.8 ± 37.9 µm) and amikacin-treated (2320.8 ± 36.1 µm) animals (t (12) = 0.727, p = 0.48) in the maximum dimension of the IC along this axis.

## Discussion

Our results revealed that rats that received daily systemic treatments of amikacin over postnatal days 7 through 16 had severe cochlear damage when they became 3-month old (Fig. 1). The drug caused a complete loss of IHCs and OHCs in the basal cochlea. It caused partial loss of IHCs and OHCs in the middle and apical cochlea with the damage being more severe in the middle than the apical turn. OHCs were affected more than IHCs by the drug. Amikacin caused partial loss of SGNs over the entire cochlea, with the extent being largest in the basal and smallest in the apical turn. Thus, our results agreed with existing findings regarding effects of aminoglycosides on the auditory sensory organ^17–20,36,37^. Such changes could severally affect neurophysiological responses to sounds^37,38^.

Despite destruction of hair cells and SGNs in the entire cochlea, an LFP elicited by intracochlear stimulation was reduced only in the ventromedial area of the IC (Figs 7 and 8). The response was enhanced rather than reduced in the dorsolateral area. This area-dependent change led to a shift of the best location of LFP response from a central to a more dorsolateral region of the IC. The change of the area that could be activated by intracochlear stimulation agreed with previous studies regarding the effect of the drug on neural activity in the IC. A study in adult rats that received systemic treatments of amikacin at the onset of hearing revealed that intracochlear stimulation could elicit immunoreactivity against *Fos* only in neurons outside the ventromedial IC^36^. Studies based on single-unit recordings revealed that newborn chinchillas treated with amikacin developed an altered topographic distribution of auditory neurons over the IC when the animals reached adulthood^29,30^. The area with neurons that could be activated by sounds was shifted toward the dorsolateral part of the IC. Specifically, neurons that could be activated by acoustic stimulation were almost completely absent in the ventromedial region. Many neurons between the dorsolateral and ventromedial regions changed their frequency tuning and became sensitive to a single frequency that was associated with the boundary between the damaged and unaffected cochlear regions.

Thus, our findings along with previous results revealed a contrast between the alterations in the IC and in the peripheral sensory organ following systemic application of amikacin. This contrast suggests that plastic changes were induced along the central pathways, which enabled neurons in the dorsolateral region of the IC (i.e., the region sensitive to low frequency sounds) to increase their “gain” in response to activation of the auditory nerve. This increase agrees with findings from other studies on central changes following cochlear damage caused by intense noises or drugs^4,6,31–34,39^. In the case when cochlear damage was produced in a chinchilla using a narrow-band intense noise, LFPs recorded in IC were enhanced at frequencies below the edge of the hearing loss and reduced at frequencies within the region of hearing loss^6,33,39^. Thus, damage caused by either amikacin or a narrow-band intense noise led to a frequency-dependent enhancement of “gain” in the IC. The possibility exists that an enhancement of “gain” in the low-frequency area of the IC is a common plastic change caused by any type of partial cochlear damage.

The latency of the negative peak of LFP was increased over the entire dorsolateral-ventromedial extent of the IC (Fig. 9). It was noted that a difference in the depth-dependence existed between drug-induced changes of peak latency and peak amplitude. For example, the mean amplitude of response was significantly increased at a depth of 0.8 mm but significantly reduced at a depth of 1.2 mm in amikacin-treated animals. In contrast, the mean latency was significantly increased at both depths in amikacin-treated animals. This finding was consistent with results from adult cats that were deafened by direct cochlear perfusion of kanamycin before hearing onset^40,41^. These animals had increased latencies of auditory brainstem responses and intracochlear stimulation-elicited single-unit responses in the IC. An increase in the latency of response, along with a modification of “gain”, can greatly affect neural processing in the IC.

Area-dependent changes of LFP in the IC in response to intracochlear stimulation were likely due to factors other than shrinkage of the structure. Although systemic applications of amikacin are known to cause shrinkage of the cochlear nuclei^37,40^, they did not cause a similar change in the IC.

Several factors might have contributed to the reduction of the LFPs in the ventromedial IC. One contributing factor might have been the destruction of SGNs in the basal cochlea. In the present study, LFPs were recorded at locations between 0.2 and 2.4 mm from the surface of the IC. These locations were within the central nucleus of IC^26^, which has neurons forming a tonotopic map^26,28,42^. While neurons in the dorsolateral area are sensitive to low frequencies and are indirectly driven by SGNs in the apical cochlea, those in the ventromedial area are sensitive to high frequencies and are indirectly driven by the basal cochlea. Essential features of this pattern are established before hearing onset^22,36,43,44^. Loss of SGNs in the basal cochlea due to drug application could certainly limit inputs to the IC and reduce the LFPs in the ventromedial area of the structure. The reduction might have also been partly due to destruction of hair cells in the basal cochlea. Previous studies have indicated that electrical currents applied to the cochlea can stimulate not only SGNs (i.e., electroneural stimulation) but also hair cells (i.e., electrophonic stimulation)^45,46^. Furthermore, the reduction of LFPs could have been dependent on central plastic changes such as alteration of synaptic density in the auditory midbrain^47^ and reduction in the number of neurons projecting from the cochlear nucleus to the IC^37^.

Factors contributing to the enhancement of intracochlear stimulation-elicited LFP in the dorsolateral region of the IC were likely related to central changes rather than damage of the hair cells and SGNs that drive the activity in this collicular region. Reorganization of neural connections within auditory pathways might have been among those central changes. As cochlear damage produced by amikacin started at the basal cochlea, a difference in the level of activity could exist between ascending fibers from basal and apical cochlear regions. This might have put neurons in the ventromedial IC at a competitive disadvantage and neurons in the dorsolateral IC at a competitive advantage in synaptogenesis. This could have resulted in an increased number of fibers innervating the dorsolateral region of the IC and an enhanced LFP response in the area. This mechanism based on “competitive innervation” has been used to explain visual central plasticity caused by sensory deprivation^48^ as well as amikacin-induced changes in the tonotopic organization in the IC^29,30,49^. The mechanism could have also contributed to the enhancement in LFP response in the dorsolateral IC in the present study.

Other factors that might have affected LFP responses in the IC likely included alterations of excitatory/inhibitory neurotransmission. It’s been revealed in the chinchilla that damage of high frequency-sensitive cochlear areas by noise overexposure or the ototoxic drug carboplatin can enhance sound-drive responses in low frequency-sensitive areas of the IC by affecting side-band inhibition^4,6,34,50^. At a molecular level, the abundance of mRNAs that encode subunits of a few neurotransmitter receptors are changed in the rat’s IC a few days following the same drug treatments as those used in the present study^20^. These receptors include the N-methyl-D-aspartate (NMDA) receptor, the α-amino-3-hydroxy-5-methyl-4-isoxazole (AMPA) receptor, and the type-A γ-aminobutyric acid (GABA_A_) receptor. Although relatively long-term effects of amikacin treatments (e.g., 3 months following treatments) on receptor expression have not been evaluated, the existence of such effects is supported by studies based on noise-induced cochlea damage^4,51,52^.

The enhancement of LFP in the dorsolateral region of the IC might have also reflected neurobiological changes in lower brainstem structures. Both “competitive innervation” and alteration of neurotransmission could have occurred in these structures. In addition, amikacin could have changed the tonotopic organization of the cochlear nucleus, a major source of excitatory projections to the IC, by reducing the physical size of the lower brainstem structure^37,40^. Furthermore, damage of hair cells in the high-frequency region of the cochlea could have increased thresholds and widened tuning curves of primary nerve fibers, which could have enhanced the sensitivity of the fibers to lower frequencies^53^. These changes in lower auditory structures could have been imparted on LFP responses in the IC in two different ways. First, responses of individual neurons in the IC could have been driven/modulated by inputs that were either directly or indirectly influenced by neurons in the lower auditory structures. Second, LFPs recorded in the IC could have included presynaptic activities generated by axon terminals of ascending fibers from neurons in brainstem structures. Contributions of spiking activities of presynaptic terminals to an LFP should be considered especially when the upper cutoff frequency of the amplifier used in the recording is high^54^. In the present study, the upper cutoff frequency was set at 5 kHz due to a relatively short time course of the LFPs. A low-impedance recording electrode was used to limit the contribution from presynaptic terminals. Relatively smooth waveforms recorded in the present study indicated that this contribution was minor if present at all.

An increase in the latency of LFP in the IC following amikacin treatments was likely dependent on central rather peripheral changes. Electrical shocks delivered to the cochlea could have activated both hair cells and SGNs^45,46^. The component of response in the IC resulting from activation of hair cells might have had a slightly longer latency than that resulting from activation of SGNs due to the time required for sensory transduction. Thus, a loss of hair cells would not lead to an increase in the latency of LFP responses recorded in the IC. Central factors that could have caused an increase in the latency of response would include reduction of synaptic efficacy and demyelination of the axon fibers along the ascending pathways from the cochlea to the IC^40,55,56^.

Changes in the central auditory system induced by a cochlear damage can develop over time^1,3,13^. It is unknown whether the results from the present study reflected a final stage of such development or simply one stage in a process of plastic change that was evolving over time. The possibility exists that the dorsolateral and ventromedial parts of the IC were at different stages of plastic change when neurophysiological responses were evaluated. The change observed in the dorsolateral part of the IC might have reflected effects of both early damage of the basal cochlea and more recent damage of the apical cochlea. Further systematic research is needed to address time courses of neurophysiological changes in the IC following cochlear damages caused by amikacin.

Knowledge about central changes following cochlear damage can help understand the prognosis of cochlear implantation and develop strategies for clinical treatment. Cochlea implants have long been used to treat deafness caused by ototoxic drugs^57^. Such treatment can enhance sound-driven activities of peripheral auditory neurons, restore the sensitivity to sounds in both adult and child patients, and facilitate successful rehabilitation^58–60^. A delay in implantation can reduce the effectiveness of the treatment due to sensory deprivation-induced progressive changes in the central auditory system^13,14^. Our results indicate that these central changes include an alteration of auditory activity in the IC. The results regarding the area-dependent change in the amplitude of a intracochlear stimulation-elicited response and the overall increase in the latency of the response should be taken into consideration in the future when new cochlear implants are designed. When our results are used to make an extrapolation for addressing amikacin-induced changes in humans, it should be kept in mind that the subjects used in the present study (rats at 3-month old) had reached sexual maturity and were close to reaching social maturity^61^.

In conclusion, cochlear damage produced by amikacin treatments at the onset of hearing affects intracochlear stimulation-elicited response in the IC when the rat reaches young adulthood. Responses in areas associated with high-frequency hearing were reduced, whereas those in areas associated with low-frequency hearing were enhanced. Latencies of responses were increased over the entire area of the IC. These alterations in neurophysiological activity should be taken into consideration in the development of cochlear implantation and other methods for dealing with hearing loss.

## Methods

### Subjects

Fourteen Wistar albino rats (*Rattus norvegicus*), including 7 normal (4 males and 3 females) and 7 amikacin-treated (5 male and 2 female) rats, were used in the present study. Rat pups were purchased from Charles River Laboratories (St. Constant, Quebec, Canada) and housed and maintained in the Carleton University Animal Care Facility. They were weaned from their mothers when they were 3-weeks old and raised under normal laboratory conditions afterwards. The animals were about 3-month old and weighed about 420 g when electrophysiological recordings were conducted. All experimental procedures were approved by the Carleton University Animal Care Committee in accordance with the guidelines and regulations of the Canadian Council on Animal Care. All experiments were performed in accordance with the relevant guidelines and regulations.

### Ototoxic drug treatment

Rat pups in the experimental group were given daily injections of amikacin (500 mg/kg, s.c.) on postnatal days 7 through 16. The rats were returned to their original litter after each injection.

### Surgical procedures and electrode placements

All procedures, including those for making cochleostomies and a craniotomy and those for conducting physiological recordings, were conducted in anesthetized animals. Anesthesia was induced by 4% isoflurane (Abbott Laboratories Ltd., Saint-Laurent, QC) and maintained by 1.5% isoflurane. Neurophysiological recordings were conducted immediately following the surgery when anesthesia was maintained by ketamine hydrochloride (20 mg/kg, i.m.) and xylazine hydrochloride (3.3 mg/kg, i.m.). Atropine sulfate (0.10 mg/kg, s.c.) was used to prevent bronchial congestion.

After a midline incision was made in the scalp, cochleostomies were made for placing stimulating electrodes in the cochlea. For this purpose, the temporal muscle was retracted to expose the external meatus of the left ear. The external meatus was then cut and the tympanic membrane and the ossicles were removed. After an opening was made in the bulla, two cochleostomies were made using a tungsten carbide drill with a tip diameter of 230 µm (Gebr. Brasseler GmbH & Co. KG., Lemgo, Germany). One cochleostomy was at the basal turn right below the stapedial artery and the other one was at the apical turn. Two Teflon-insulated platinum/iridium (Pt-Ir: 90-10) microelectrodes with a diameter of 127 µm (MicroProbes, Gaithersburg, MD) were inserted into the cochlea through the two cochleostomies, respectively, to stimulate the entire cochlea. The impedance between the two electrodes was within the range of 5 to 7 kΩ. The openings of the cochleostomies were sealed by small pieces of soft tissue and the electrodes were fixed in place with Vetbond tissue adhesive (3 M Health Care, MN).

A small craniotomy was made over the right cerebral hemisphere for placing a recording electrode into the IC. The occipital cortex was aspirated to expose the underlying IC. The electrode was a single-barrel glass pipette filled with 3 M potassium chloride (tip diameter ~20.0 µm, impedance ~200 kΩ). The electrode was in the coronal plane at a 30-degree angle relative to the sagittal plane. It was first placed on the surface of the IC under visual guidance and then slowly lowered into the IC using the micromanipulator of a Model 900 stereotaxic instrument (David-Kopf Instruments, Tujunga, CA). A reference electrode was placed in subcutaneous tissue within the surgical area.

### Electrical stimulation and neurophysiological recording procedures

Electrical stimuli were generated using a S11 dual output digital stimulator (Grass Instrument Company, Guincy, MA) and delivered to the intracochlear stimulating microelectrodes through a SIU5 stimulus isolation unit (Grass Instrument Company, Guincy, MA). The stimuli were trains of 100 µs pulses presented at a 1/s rate.

Neural signals were amplified using an EX4-400 differential amplifier (Dagan Corporation, Minneapolis, MN). The low and high cut-off frequencies of the bandpass filter of the amplifier were set at 1 Hz and 5 kHz, respectively. The signals were registered at a sampling rate of 100 kHz and averaged using a Nicolet Benchtop Waveform Acquisition System 400 (Thermo Nicolet, Madison, WI). At each recording locus, LFP responses were obtained at various levels of intracochlear electrical stimulation. At each level of stimulation, an averaged LFP response was obtained using signals elicited by 10 stimuli.

### Histological procedures

At the completion of each neurophysiological experiment, the rat was euthanized with sodium pentobarbital (120 mg/kg, i.p.) and transcardially perfused with heparinized saline (20 units/ml in 0.9% NaCl) followed by 4% paraformaldehyde in 0.1 M phosphate buffered saline (pH = 7.4). The brain was extracted and postfixed in the same fixative for 24 h. It was then cryoprotected in 25% sucrose in 0.1 M phosphate buffered saline (pH = 7.4) and sectioned using a cryostat in the coronal plane at a 40-μm thickness. Sections were stained with cresyl violet.

Following transcardial perfusion, the cochlea was extracted under a dissecting microscope and postfixed in 4% paraformaldehyde at 4 °C for 24 h. After decalcification in 5% EDTA for 48 h, it was imbedded in paraffin using standard histological procedures. Cross sections of the cochlea were made using a sliding microtome at a 4-μm thickness to reveal both hair cells and SGNs. The sections were stained with hematoxylin and eosin.

Histological results, including both those from the IC and the cochlea, were observed under an Eclipse E1000M light microscope (Nikon, Tokyo, Japan).

### Data analysis

Hair cell survival rates were obtained for different cochlear regions using the method developed by Kinoshita and colleagues^62^. Surviving IHCs and OHCs were counted separately for the apical, middle, and basal regions of the cochlea. To ensure that cells counted for a specific cochlear region did not contain any cells from an adjacent region, sections from the top 1/4 of the middle and basal turns were not used in the counting. A minimum of 12 sections were selected randomly from each of the three cochlear regions for cell counting.

The morphology of each hair cell in the sections was examined. Only a cell that did not show any damage of the cell body, nucleus, or cuticular plate was considered to be a surviving cell. Examinations indicated that each tissue section from a normal animal contained three OHCs and one IHC. No OHCs or IHCs from these sections showed any signs of cell damage. Thus, it was assumed that the destruction of hair cells in amikacin-treated animals was caused by drug application rather than a sectioning process. The total number of IHCs (including both surviving and damaged cells) in a set of sections from a drug-treated animal would be expected to equal the number of sections. The total number of OHCs (including both surviving and damaged cells) would be expected to equal 3 times the number of examined sections. Survival rates of IHCs and OHCs were calculated for each turn of a cochlea based on these expectations:

$${\rm{IHC}}\,{\rm{survival}}\,{\rm{rate}}=\frac{{\rm{Number}}\,{\rm{of}}\,{\rm{intact}}\,{\rm{IHCs}}\,{\rm{in}}\,{\rm{examined}}\,{\rm{sections}}}{{\rm{Number}}\,{\rm{of}}\,{\rm{examined}}\,{\rm{sections}}}\times 100$$

$${\rm{OHC}}\,{\rm{survival}}\,{\rm{rate}}=\frac{{\rm{Number}}\,{\rm{of}}\,{\rm{intact}}\,{\rm{OHCs}}\,{\rm{in}}\,{\rm{examined}}\,{\rm{sections}}}{{\rm{Number}}\,{\rm{of}}\,{\rm{examined}}\,{\rm{sections}}\times 3}\times 100$$

Survival rates of IHCs and OHCs were compared among the three regions of the cochlea. In this comparison, a logistic regression with interaction terms (i.e., IHC vs. OHC for apical, middle, and basal turns) fitted by generalized estimating equations was used to account for repeated measurements within the same cochlear region. The SAS V9.4 software (SAS Institute, Cary, NC) was used for the comparison.

Three mid-modiolar sections were randomly chosen from each cochlea for counting of intact SGNs. Each of these sections contained three areas of SGNs that were in the top, lower ¾ of the middle turn, and lower ¾ of the basal turn of the cochlea, respectively. The number of SGNs and the area of Rosenthal’s canal were measured for each area using the Photoshop CS4 software (Adobe Systems, San Jose, CA). A mean density of SGN was calculated for each of the three parts of the cochlea using measurements from the three representative sections.

Averaged LFP waveforms saved in the Nicolet Benchtop Waveform Acquisition System were exported into DPlot version 2.2.9.3 (HydeSoft Computing LLC, Vicksburg, MS) for offline analysis. The amplitude and latency of the negative peak of an LFP waveform were measured.

To determine whether amikacin treatments caused shrinkage of the IC, the size of the structure was measured for all the normal and amikacin-treated animals using an axis within the coronal plane at a 30° angle relative to the sagittal plane (Fig. 2). The maximum distance between two points where the axis intersected the dorsolateral and ventromedial edges of the structure was obtained.

Statistical analyses that did not involve logistic regression were performed using the SPSS 17.0 software (IBM Corporation, Armonk, NY).

## Data Availability

The datasets generated and analysed during the current study are available from the corresponding author on reasonable request.
